# Single Cell Map of Human Azoospermia Testis Caused by Cyclophosphamide Chemotherapy

**DOI:** 10.1038/s41597-024-02938-5

**Published:** 2024-02-02

**Authors:** Jian Cao, Xueheng Zhao, Zailong Qin, Shanshan Lv, Lin Du, Zhizhong Liu, Liqing Fan, Hao Bo

**Affiliations:** 1 Department of Urology, Hunan Cancer Hospital, the Affiliated Cancer Hospital of Xiangya School of Medicine, Central South University, Changsha, Hunan China; 2 NHC Key Laboratory of Human Stem Cell and Reproductive Engineering, Institute of Reproductive and Stem Cell Engineering, School of Basic Medical Science, Central South University, Changsha, Hunan China; 3 Laboratory of Genetics and Metabolism, Maternal and Child Health Hospital of Guangxi Zhuang Autonomous Region, Guangxi Birth Defects Research and Prevention Institute, Nanning, Guangxi China; 4 Department of Blood Transfusion, the Third Xiangya Hospital of Central South University, Changsha, Hunan China; 5 Clinical Research Center for Reproduction and Genetics in Hunan Province, Reproductive and Genetic Hospital of CITIC-Xiangya, Changsha, Hunan China

**Keywords:** Infertility, Spermatogenesis

## Abstract

Chemotherapeutic drugs will affect the process of spermatogenesis. However, most current studies on the effects of chemotherapeutic drugs on spermatogenesis are based on mouse models, with a shortage of human body evidence. In addition, the mechanism of chemotherapeutic drugs causing spermatogenesis disorder is not clear. Therefore, we have collected the testicular tissues of an inguinal-lipoma patient whose testes were resected after chemotherapy and a patient who had normal spermatogenesis disorder and underwent single-nucleus RNA sequencing (snRNA-Seq). After quality control, we obtained a total of 27,957 high-quality cells, including 18,612 normal cells and 9,345 drug-treated cells, which were all used in analyzing the mechanism of chemotherapeutic drugs causing spermatogenesis disorder. This study has provided data resources and references for exploring the mechanism of chemotherapeutic drugs causing spermatogenesis disorder with the insight of protecting the spermatogenic abilities of male tumor patients receiving chemotherapy.

## Background & Summary

Spermatogenesis is a complex and delicate process, which starts in the spermatogonial stem cells (SSCs) at the bottom of the seminiferous tubule base, with mature male gametes eventually generated. Some SSCs are in a state of “resting”, while others are in a state of “proliferating”. On the one hand, the stem cell bank is maintained through proliferation of stem cells. On the other hand, these cells undergo a series of differentiation processes, changing from spermatogonia to primary spermatocytes and then to secondary spermatocytes, which develop into spermatids that finally evolve into sperms. The whole process of spermatogenesis is regulated by such surrounding cells as Sertoli cells, Leydig cells, and peritubular myoid cells, all of which interact with each other in their functions^1^. Bulk RNA sequencing can reflect the average expression level of genes of various cell types in the sample. However, it can not effectively present the gene expression changes of different cells or the gene expression characteristics of various cell subtypes in the sample, without revealing the heterogeneity of cells. Single-cell RNA sequencing (scRNA-Seq) can capture the gene expression of each cell, and can be used to effectively analyze the heterogeneity of cell samples. Therefore, it is possible to explore the mechanism of human spermatogenesis in greater detail.

Testicular tissues, especially SSCs and spermatogonia in the states of proliferation and differentiation and spermatocytes in meiosis during the process of spermatogenesis, are very sensitive to chemotherapy. After chemotherapy, the sperm quality of patients will drop drastically first and then progressively for months. This indicates that anticancer drugs have a long-term effect on SSCs^2^. Animal experiments show that chemotherapy can reduce the number and proliferation activities of SSCs in rats, and meanwhile, spermatocytes are sensitive to the cytotoxicity effects of anticancer drugs. In addition, chemotherapy will lead to the vacuolation of seminiferous tubules and the increased apoptoses of spermatogonia and spermatocytes, with decreased diameters of seminiferous tubules and shrunk testes of rats^3^. In terms of mechanism, many chemotherapeutic drugs will lead to an increase of oxidative stress in testis tissues, which is related to the down-regulation of antioxidant enzymes. And antioxidant enzymes are necessary to prevent the excessive formation of reactive oxygen species (ROS). Increased oxidative stress could result in DNA damage among germ cells and eventually lead to the demise of germ cells^4,5^. For example, some research has shown that cyclophosphamide can induce the apoptoses of spermatogonia and spermatocytes^6^. Although other somatic cells in the testes have good tolerance to chemotherapeutic drugs, their functions will be damaged to varying degrees. Also, cyclophosphamide used in adult mice will lead to disordered protein expressions of Sertoli cells^7^. In summary, existing research on the influences of chemotherapeutic drugs on spermatogenesis is mostly based on models of mice or rats. However, the spermatogenesis processes of lower rodents investigated are significantly different from the spermatogenesis processes of human beings. Therefore, with the animal models of lower rodents, the spermatogenesis mechanism of human beings can not be simulated completely and precisely. Thus, it is of particular importance to use human samples in such research.

In this study, we have collected the testicular tissues of tumor patients treated with cyclophosphamide chemotherapy and normal people and generated the testicular single-cell map of chemotherapy-treated patients by using snRNA-Seq. Therefore, this study has provided a reference for exploring the influences of chemotherapeutic drugs on various germ cells and somatic cells in human testicular tissues and developing new methods for preventing spermatogenic cell damage caused by chemotherapy.

## Methods

### Ethical statement

The implementation of this project has been approved by the Ethics Committee of Hunan Cancer Hospital, and all patients involved in this project have signed an informed consent form and consented to data sharing.

### Collection and processing of testicular tissue samples

Human testicular samples used in this study were collected by the surgical urology department of Hunan Cancer Hospital. Among them, samples in the normal control group were taken from a patient with the scrotal epidermoid cyst, and samples in the chemotherapy group were taken from a cyclophosphamide chemotherapy-treated patient with inguinal lipoma (Supplementary table 1). This inguinal-lipoma patient was treated with orchiectomy because it was suspected that the tumor had invaded his testes, which was finally denied by the pathology analysis. Another patient was treated with orchiectomy because he was suspected to have suffered from testicular tumor, with pathology analysis showing that he had actually contracted an epidermoid cyst of scrotum. Samples in the normal control group presented normal testicular spermatogenesis, while samples in the chemotherapy group presented failed testicular spermatogenesis.

Upon the receipt of fresh testicular samples, they were washed in a saline solution immediately to remove blood stains and then dried out with absorbent paper. Then, samples with a size of about 5 mm × 5 mm × 5 mm were taken with surgical instruments and put in liquid nitrogen for quick freezing and later use.

### HE staining

Testicular tissue sections were baked in an oven (Yidi, China) at 65 °C for 1 h. Sections were deparaffinized with xylene, rehydrated with graded ethanol, and stained with hematoxylin (Beyotime, China) for 8 min at room temperature. Then, after rinsing with running water and staining with eosin (Beyotime, China) for 3–10 s at room temperature, these sections were rinsed with running water for about 30 s (it is preferred that eosin is cleaned off from the slides). The slides were rapidly passed through 70%, 80%, 95% anhydrous ethanol in sequence, transparentize in xylene 3 times for 2 min each time, and then sealed with neutral resin (Sinopharm, China). The results show that normal testicular tissues without chemotherapy present normal spermatogenesis and testicular tissues after cyclophosphamide chemotherapy present impaired spermatogenesis (Supplementary Fig. 1)

### Quality control of frozen testicular samples

According to the instructions of the Trizol reagent, total RNA was extracted from frozen testis tissues through the grinding method and Trizol method, and the RNA content was quantified with Nanodrop 2000 (Thermo Fisher Scientific, MA, USA). The quality and integrity of RNA were tested with Agilent 2100/GX Bioanalyzer. Samples with RNA integrity number (RIN) greater than 7.5 were used for subsequent library construction and sequencing.

### Testicular single-cell nucleus isolation

Firstly, a tissue sample was transferred to a grinder containing 2 mL of pre-cooled homogenization buffer (containing 20 mM of Tris pH8.0, 500 mM of sucrose, 0.1% NP-40, 0.2U/μL RNase inhibitor, 1x Protease inhibitor cocktail, 1% bovine serum albumin (BSA), and 0.1 mM of DTT), and then the sample was left on ice for 3–5 minutes. After the tissue sample was fully infiltrated, tissue blocks were ground with a grinding rod (about 10–15 times) until their resistance disappeared. Then, a microscope was used to check whether the nuclei were fully released and whether the suspension background was clean. Then, the tissue homogenate was filtered with 70 µm and 40 µm filters, and then, the collected filtrate was transferred to a 15 mL centrifuge tube at 4°C and was centrifuged at 500 g for 5 minutes. After its supernatant was removed, the filtrate was washed twice with 1 mL of Wash Buffer to remove impurities. A proper volume of Blocking Buffer (containing 1% BSA and 0.4U/μL RNase inhibitor in 1x PBS) was used to resuspend the precipitate. After the filtrate was evenly mixed, a microscope was used to check the background of the nucleus suspension and the nucleus integrity, and samples with no background impurities and a nucleus integrity degree greater than 85% were selected for follow-up experiments.

### Library construction and sequencing of single-nucleus RNA

A DNBelab C4 device (MGI) was used to capture single nucleus RNA based on the instructions of the MGI DNBelab C series Reagent Kit (MGI, 940-000047-00). Simply put, such procedures as droplet encapsulation, demulsification, mRNA capture, reverse transcription, cDNA amplification, and purification of single-cell nucleus were carried out with the DNBelab C4 device to establish the single nucleus RNA sequencing library with barcodes from the single-cell nucleus suspension. Then, cDNA was cut into 250–400 bp, and an index sequencing library was constructed according to the instructions. Also, Qubit ssDNA Assay Kit (Thermo Fisher Scientific) and Agilent Bioanalyzer 2100 were used to quantify the library. Finally, DIPSEQ T1 sequencing platform was used to perform the sequencing of the library.

### Raw data processing of snRNA-Seq

The DNBelab C Series scRNA-analysis software (https://github.com/MGI-tech-bioinformatics/DNBelab_C_Series_HT_scRNA-analysis-software) was used in filtering, comparing, and quantifying the sequencing data of single-nucleus RNA, with detailed procedures carried out based on the previously published research of MGI team.

### Filtering, dimensionality reduction, clustering, and cell type annotation of snRNA-Seq data

Seurat package (version 4.2.1, https://www.r-project.org/) in R software was used to analyze the single-cell expression data. First, after importing the expression data, we used the subset function in the Seurat package to filter the data with unique feature counts greater than 400 and less than 7,000 and mitochondrial gene counts lower than 15% as the threshold. After the quality control, cells were standardized with the “NormalizeData” function. Then, a UMAP analysis was performed on the top 2,000 highly variable genes and the top 30 principal components selected. Finally, a cluster analysis was conducted under the condition of resolution = 1.5. Cell types were annotated manually based on the marker provided in the published literature.

### Enrichment analysis of differentially expressed genes

The “FindAllMarkers” function of Seurat package was used to identify the differentially expressed genes. Genes with LogFC threshold >0.5 were identified as differentially expressed genes. An enrichment analysis of differentially expressed genes using the “enrichCluster” function in ClusterGVis package was performed, with the top 10 enriched terms of each cell type displayed.

## Data Records

All the original data have been uploaded to the Genome Sequence Archive (GSA) database, with the number of HRA005608 (ngdc.cncb.ac.cn/gsa-human). Those processed sample expression matrix data have been uploaded to the Figshare database (10.6084/m9.figshare.24198027)^8^.

## Technical Validation

**Fig. 1:**
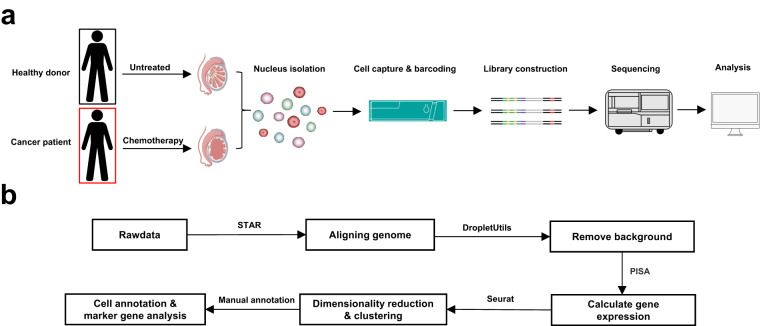
Workflow of snRNA-Seq and data analysis. (**a**) Flow chart of testicular single-nucleus RNA sequencing. We collected testicular tissue samples from a patient who had not undergone any treatment and a patient who had undergone cyclophosphamide chemotherapy. After freezing the tissue samples in liquid nitrogen, we extracted single cell nuclei from these samples and then used the DNBelab C4 device to capture and label mRNA. Next, sequencing libraries were constructed according to the manufacturer’s instructions. Finally, RNA sequencing using the DIPSEQ T1 sequencing platform was performed. (**b**) Flow chart of snRNA-Seq data analysis. We first used the STAR tool to align reads to the genome reference, and then used the DropletUtils tool to remove background droplets. Finally, PISA was used to generate a gene expression matrix. A downstream analysis of snRNA-Seq data using the Seurat package was carried out, and cell type annotation was completed in a manual way.

We collected testis tissues of patients treated with cyclophosphamide chemotherapy and without any drug chemotherapy. After the extraction of the single-cell nuclei, a single-nucleus RNA sequencing was performed with the DNB Lab C4 platform (Fig. 1a). After the sequencing, STAR, DropletUtils, and PISA were used to perform the genome alignment, background removal, and gene expression quantification, and the Seurat package was used to carry out the downstream analysis (Fig. 1b). The detailed alignment information of each sample’s sequencing data is listed in Table 1. It can be seen that the genome alignment rate of each sample’s measured data is above 95%, and the Q30 Bases in Barcode range from 91.4% to 96.5%, with the total reads number of each sample higher than 437 M. Also, the quality statistics of each sample’s detailed sequencing data are listed in Table 1. It can be seen that the number of captured cells in each sample ranges from 2,752 to 7,177, the median number of cell genes in each sample ranges from 2,180 to 4,186, and the total number of captured genes in each sample is higher than 42,000 (Table 2). In summary, through the single-nucleus RNA sequencing, we have obtained the high-quality transcriptome data of single cell nuclei of testicular tissues collected from patients treated with chemotherapy.

**Table 1:** Quality control of raw snRNA-seq data.

Sample	Reads	Q30 Bases in Barcode	Q30 Bases in RNA Read	Q30 Bases in UMI	Confident Mapping to Genome	Confident Mapping to Transcriptome
N1	730,549,316	91.40%	93.40%	89.20%	95.10%	68.10%
N2	774,817,596	91.90%	92.80%	90.00%	95.80%	70.00%
N3	697,890,954	91.50%	93.30%	89.40%	96.10%	70.30%
T1	572,709,353	96.50%	93.10%	93.60%	97.50%	76.00%
T2	587,051,219	96.30%	93.00%	93.60%	97.20%	74.80%
T3	437,790,587	95.90%	92.90%	92.90%	97.50%	73.30%

**Table 2:** Quality control of cell-based snRNA-Seq data.

Sample	Estimated number of cell	Mean reads per cell	Median UMI counts per cell	Median genes per cell	Total genes detected	Mitochondria ratio
N1	7,177	38,264	4,691	2,180	47,185	4.16%
N2	6,838	38,615	6,364	2,715	46,658	4.44%
N3	6,667	31,510	6,542	2,862	46,099	4.05%
T1	3,645	90,060	5,579	2,992	43,068	3.19%
T2	2,752	105,878	9,502	4,186	42,593	3.20%
T3	3,308	73,296	5,674	3,054	43,087	3.08%

**Fig. 2:**
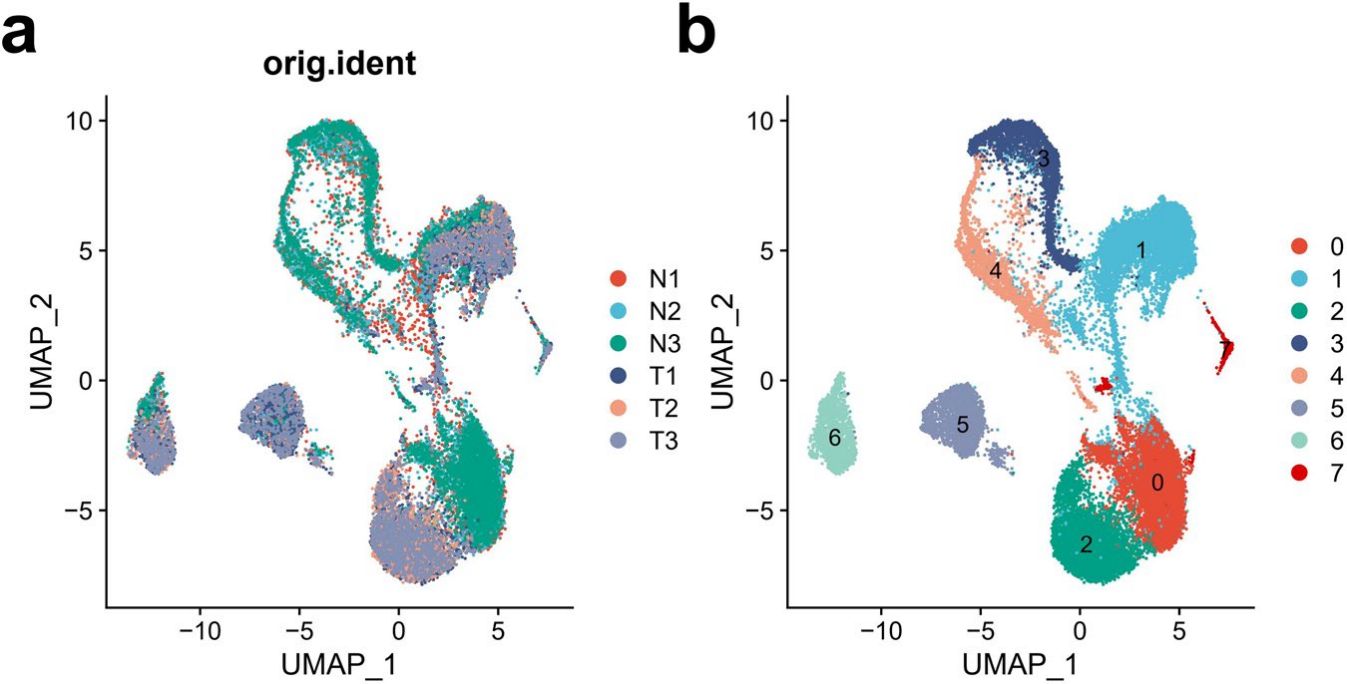
Cell clustering U-map of snRNA-Seq samples. (**a**) Comparison of untreated and cyclophosphamide-treated testes through U-Map analysis. (**b**) U-Map overview of cell clustering, with eight clusters in total. N: Normal; T: Treat.

In order to further verify the quality of the sequencing data of single-nucleus RNA, a cell clustering analysis was performed. We found that, from the perspective of sample sources, there are significant differences in the distribution of some cell clusters in different groups (Fig. 2a). According to the clustering algorithm, all single cells are divided into eight clusters numbered from 0 to 7, and every two clusters have a clear boundary between them. It shows that the sequencing data of single-nucleus RNA in this study present relatively good quality, which can fully satisfy the requirements of single-cell clustering (Fig. 2b).

**Fig. 3:**
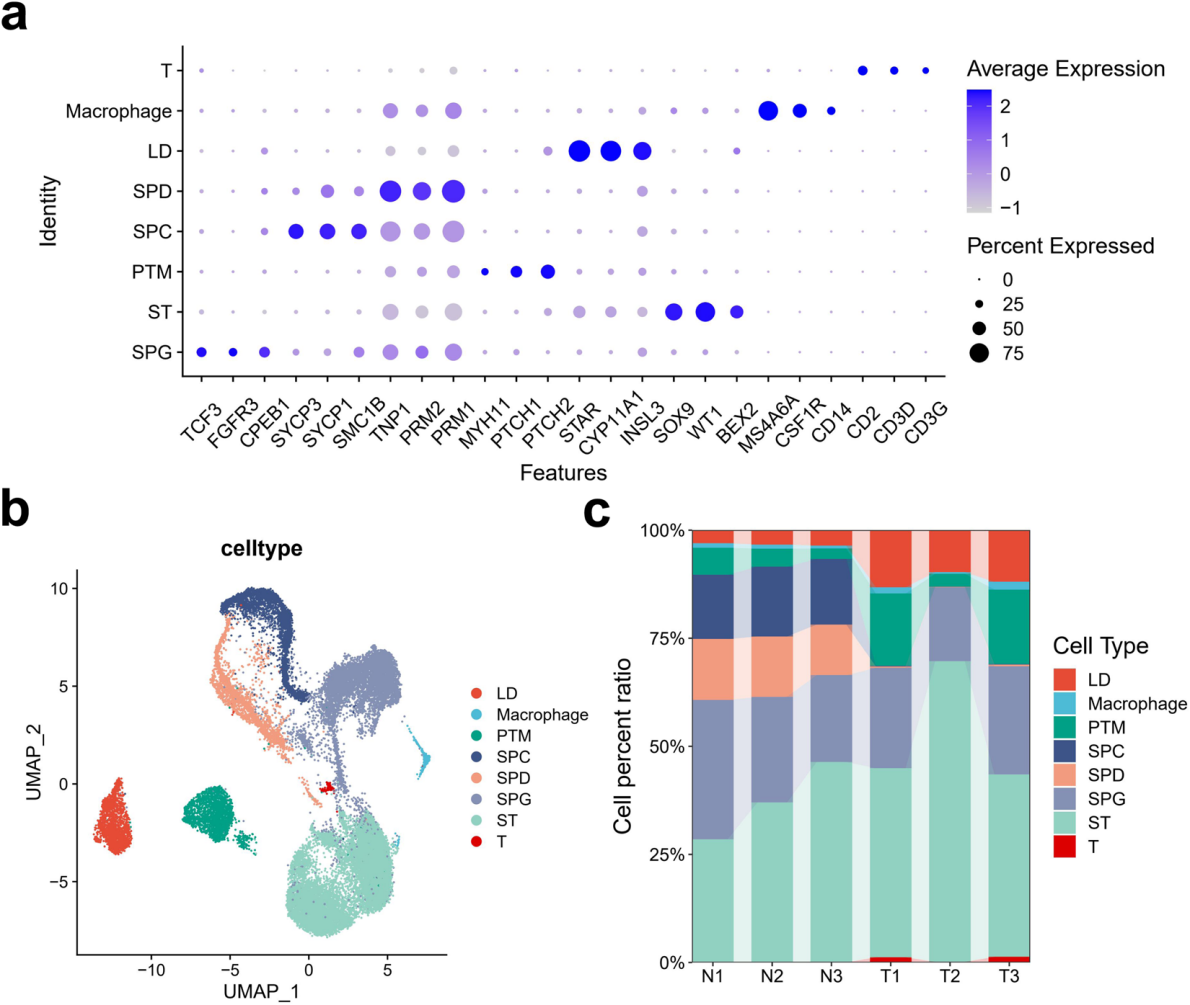
Cell type annotation of testicular snRNA-Seq data. (**a**) Dotplot-displayed marker genes of cell types identified in snRNA-Seq data. T cell: CD2, CD3D, CD3G. Macrophage: MS4A6A, CSF1R, CD14. LD: STAR, CYP11A1, INSL3. SPD: TNP1, PRM2, PRM1. SPC: SYCP3, SYCP1, SMC1B. PTM: MYH11, PTCH1, PTCH2. ST: SOX9, WT1, BEX2. SPG: TCF3, FGFR3, CPEB1. (T: T cell; LD: Leydig cell; ST: Sertoli cell; SPG: Spermatogonia; SPC: spermatocyte; SPD: spermatid; PTM: Peritubular myoid cell). (**b**) U-Map and clustering analysis of snRNA-Seq data of cyclophosphamide-treated and untreated testes, with cells colored based on their identities. (**c**) Comparison between stack diagram-displayed proportion changes of cell types in the cyclophosphamide-treated group and the normal group. N: Normal; T: Treat.

Furthermore, based on the marker gene information provided in the published literature, we displayed the marker genes of possible cell types in the samples. It can be seen that the marker genes of such testicular germ cells as spermatogonia (SPG), spermatocyte (SPC) and spermatid (SPD) and such testicular somatic cells as Sertoli cell (ST), Leydig cell (LD), and peritubular myoid cell (PTM) all present specific expressions. In addition, we also identified the specific expressions of marker genes of a small number of immune cells such as T cells and macrophages (Fig. 3a). From images, it can be easily seen that there are clear boundaries among these different cell types, indicating a good clustering effect (Fig. 3b). In terms of the proportions of cell types, it has been found that after chemotherapy, SPG are the only germ cells left in the testes of patients treated, and basically, there are few SPC and SPD left in their testes. Interestingly, it has been found that there is a higher proportion of T cells (Fig. 3c) in the testes of chemotherapy-treated patients, indicating that chemotherapy could result in imbalanced testicular immune homeostasis.

**Fig. 4:**
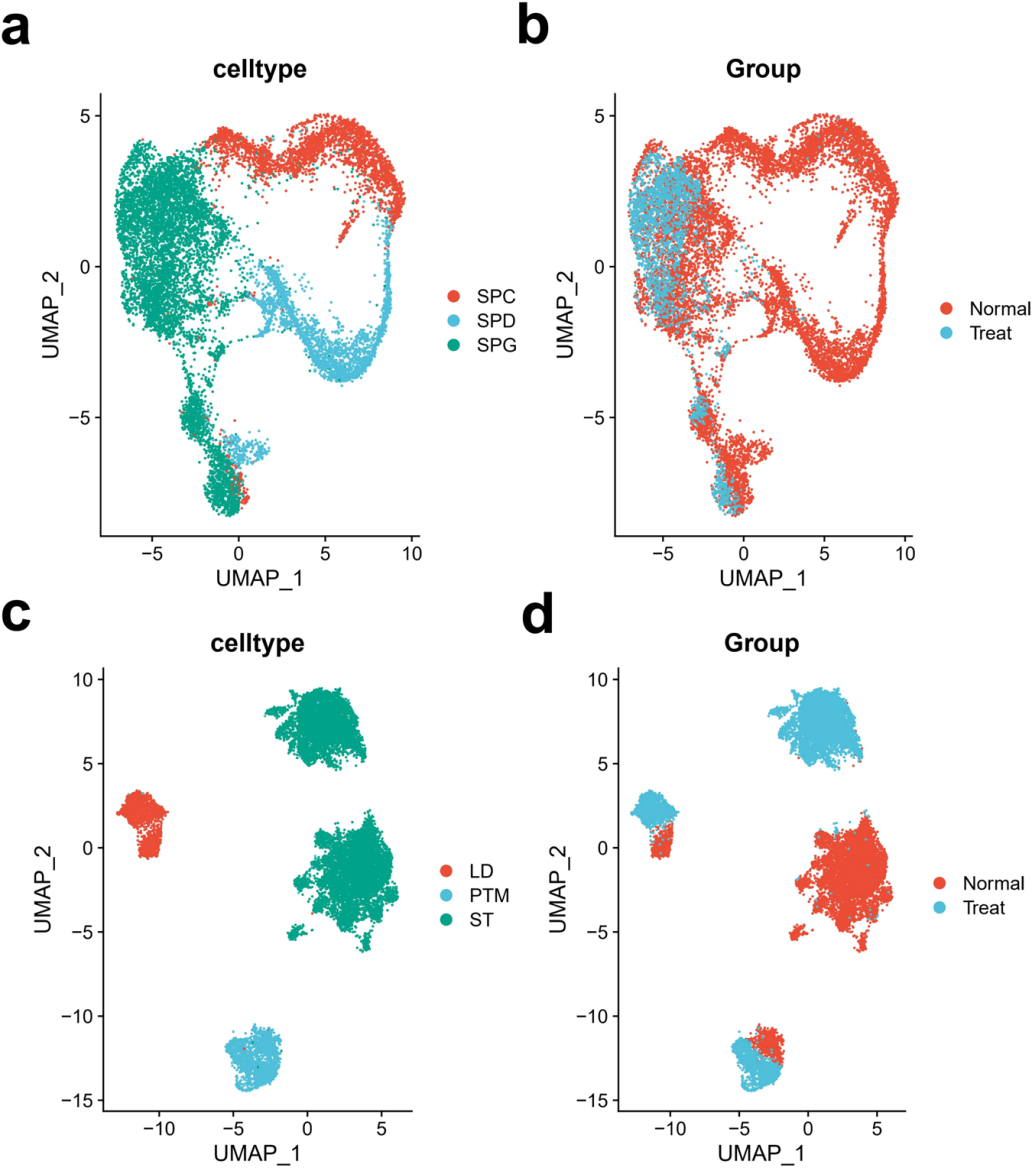
SnRNA-seq-revealed cluster changes of testicular cells after cyclophosphamide chemotherapy. (**a**) Re-clustering and subpopulation annotation of testicular germ cells. (**b**) Comparison of spermatogonia in tissue samples from untreated and cyclophosphamide-treated testes through U-Map analysis, with cells colored based on their cyclophosphamide treatment. (**c**) Re-clustering and subpopulation annotation of testicular somatic cells. (**d**) Focused analysis of ST, LD and PTM cells, which are colored based on their cyclophosphamide treatment.

Studies based on mouse models show that chemotherapy can affect the protein expressions of testicular germ cells and testicular somatic cells^9^. However, how chemotherapeutic drugs affect spermatogenesis at the transcriptome level is not clear. Again, we performed clustering on the testicular germ cells and testicular somatic cells extracted separately. The results show that testicular germ cells of samples in the chemotherapy group and the normal group exhibit good clustering performances (Fig. 4a,b). Interestingly, those three testicular somatic cells in the two groups exhibit significantly different clusterings. Especially, those ST of samples in the chemotherapy and normal groups cluster into two different clusters (Fig. 4c,d). It indicates that chemotherapy has a more drastic effect on testicular somatic cells at the transcriptome level. Furthermore, chemotherapy exerts a strongest effect on ST. Therefore, it is of particular importance to protect ST to reduce the spermatogenic injury caused by chemotherapy.

**Fig. 5:**
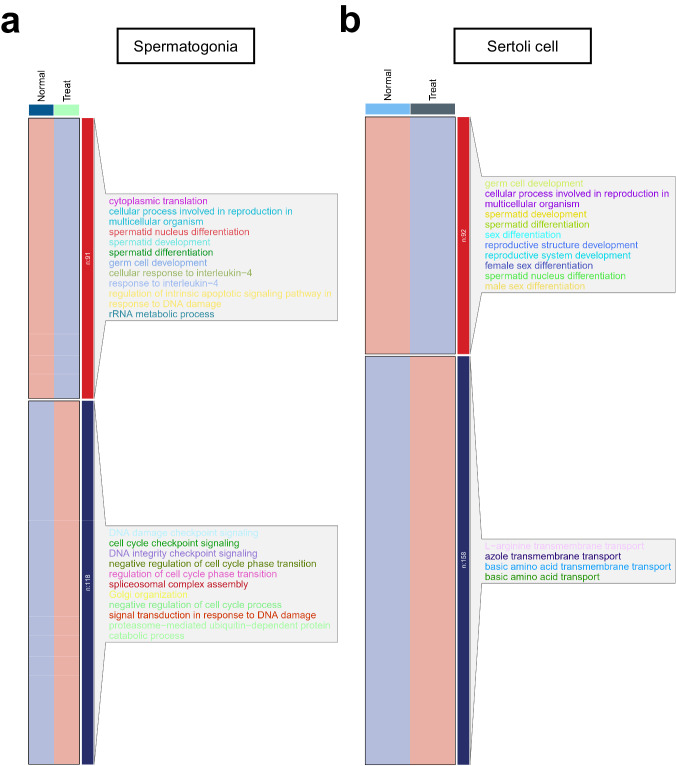
SnRNA-Seq-revealed gene expression and signaling pathway changes of germ and somatic cells after chemotherapy. (**a**) Differentially expressed genes and GO enrichment analysis in SPG after cyclophosphamide chemotherapy. One hundred and eighteen up-regulated genes and ninety-one down-regulated genes were identified in SPG. (**b**) Differentially expressed genes and GO enrichment analysis in ST after cyclophosphamide chemotherapy. One hundred and fifty eight up-regulated genes and ninety-two down-regulated genes were identified in ST.

In order to further investigate the effect of chemotherapy on testicular genes at the transcription level, a differential gene analysis on the cells in the two groups was conducted in this study. Given the fact that germ cells in the chemotherapy group are mainly SPG and somatic cells present the most pronounced changes in ST, a subsequent analysis was conducted on these two types of cells. One hundred and eighteen significantly up-regulated genes and ninety-one significantly down-regulated genes in SPG were identified (Fig. 5a). Also, one hundred and fifty eight significantly up-regulated genes and ninety-two significantly down-regulated genes were identified in ST (Fig. 5b). Those significantly up-regulated genes in SPG were mainly enriched under such terms as “DNA damage checkpoint signaling” and “cell cycle checkpoint signaling”. Meanwhile, those significantly down-regulated genes in SPG were mainly enriched under such terms as “cytoplasmic translation” and “spermatid development” (Fig. 5a). Moreover, we found that genes significantly up-regulated in ST were enriched in the forms of “L-arginine transmembrane transport”, “azole transmembrane transport” and so on. Lastly, those significantly down-regulated genes in ST were mainly enriched under such terms as “germ cell development” and “spermatid development” (Fig. 5b).

## Usage Notes

In this study, we generated the testicular single-nucleus RNA map of cyclophosphamide chemotherapy-treated patients for the first time. The data obtained in this study can be used to explore the pathogenic mechanism of spermatogenesis disorders of chemotherapy-treated patients and clarify the interactions among different cell types in the process of spermatogenesis disorder. Also, this study has provided data resources for developing treatment methods to reduce or avoid the impairment of male fertility caused by chemotherapeutic drugs. However, there are still some limitations in this study. These sequencing data obtained are only based on the testicular single-nucleus RNA map of cyclophosphamide chemotherapy-treated patients, and it is not clear whether these data can reflect the gene expression changes among all testicular cell types caused by other chemotherapeutic drugs. Moreover, collecting testicular samples from patients after chemotherapy taking the same chemotherapy drugs are extremely difficult. Therefore, no sufficient samples were collected in this study. More samples should be used in future studies to validate our findings.

### Supplementary information

Supplementary Table 1

Supplementary Figure 1

## Data Availability

All R codes used in this paper have been uploaded to the GitHub (https://github.com/zhaoxueheng/chemical-treatment) database.
