# Supplementary material for: Single Cell Map of Human Azoospermia Testis Caused by Cyclophosphamide Chemotherapy
Source: Sci Data. 2024 Feb 2;11:163. doi: 10.1038/s41597-024-02938-5 (PMC10837125; doi:10.1038/s41597-024-02938-5)
Supplement: Supplementary file 2 — Supplementary Figure 1 [file 41597_2024_2938_MOESM2_ESM.pdf]

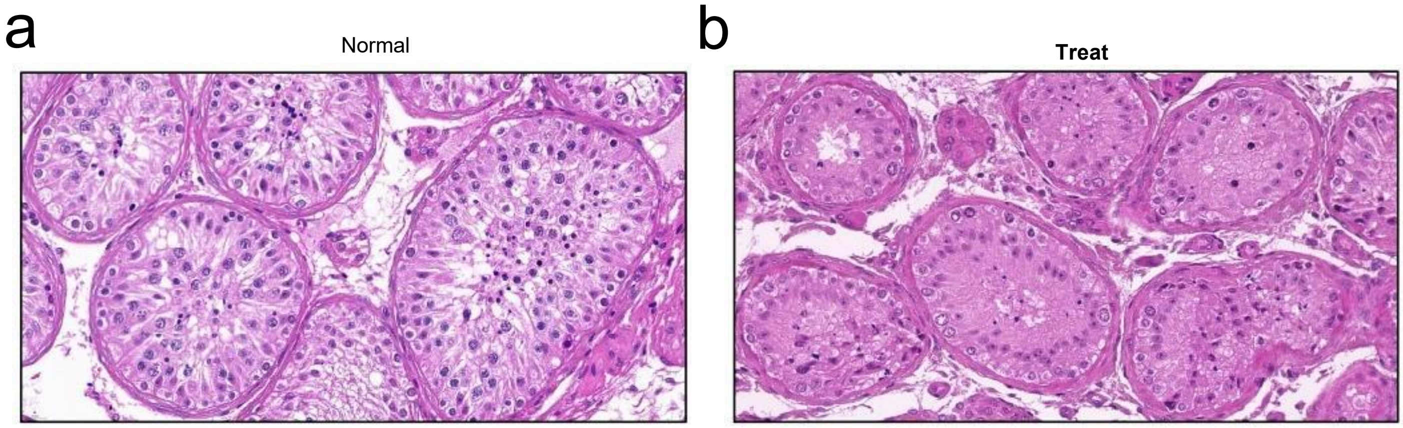

Supplementary Fig 1. HE staining of patients' testes.
A. HE staining of normal testicular samples without chemotherapy.
B. HE staining of cyclophosphamide-treated testes.
